# Depression was associated with apolipoprotein E ε4 allele polymorphism: A meta-analysis

**DOI:** 10.22038/ijbms.2018.30825.7436

**Published:** 2019-02

**Authors:** Wei-wei Wang, Xiao-lei Liu, Ye Ruan, Lin Wang, Tian-hao Bao

**Affiliations:** 1 The Second Affiliated Hospital of Kunming Medical University, Kunming City, Yunnan Province, PR China; 2 The First Affiliated Hospital of Kunming Medical University, Kunming City, Yunnan Province, PR China; 3 Mental Health Center of Kunming Medical University, Kunming City, Yunnan Province, PR China

**Keywords:** Apolipoprotein E, Depression, Meta-analysis, Polymorphism, ε4 allele

## Abstract

**Objective(s):**

Previous studies have reached different conclusions regarding an association between apolipoprotein E (APOE) polymorphisms and depression. This meta-analysis was designed to clarify these controversies.

**Materials and Methods:**

Literatures were identified reviewing the national and international databases. The eligible articles for meta-analysis were determined by quality assessment and implementation of inclusion/exclusion criteria. This meta-analysis was performed using Review Manager 5.2 software. The odds ratios (ORs) with corresponding 95% confidence interval (CIs) were calculated using a fixed effects model. Funnel plots and Egger’s regression tests were used to assess the publication bias.

**Results:**

A total of nine studies that met the inclusion criteria were identified by performing a comprehensive search on the association between APOE polymorphisms and depression. *APOE* ε4 allele was significantly associated with depression (allele: OR=1.36, 95%CI=1.11-1.66, *P*=0.003; dominant: OR=1.34, 95%CI=1.06-1.68, *P*=0.001; recessive: OR= 1.11, 95%CI =0.45-2.76, P=0.82). HAMD scores were higher in depression patients with-*APOE* ε4 genotype than who without-*APOE* ε4 genotype (OR=0.96, 95%CI=0.16-1.76, *P*=0.02).

**Conclusion:**

*APOE* ε4 allele increased the depression risk; depressive patients carrying *APOE* ε4 allele had more severe depressive symptoms.

## Introduction

Depression is a serious mental disorder affecting human health that is characterized by mood or emotional dysfunction (1). The World Health Organization speculated that by 2020, depression will become one of the main reasons that individuals are unable to work (2). The depression incidence rate has risen in recent years. The current prevalence of depression is 3-5%, it accounts for the second-highest economic burden of disease (3, 4).

**Figure 1:**
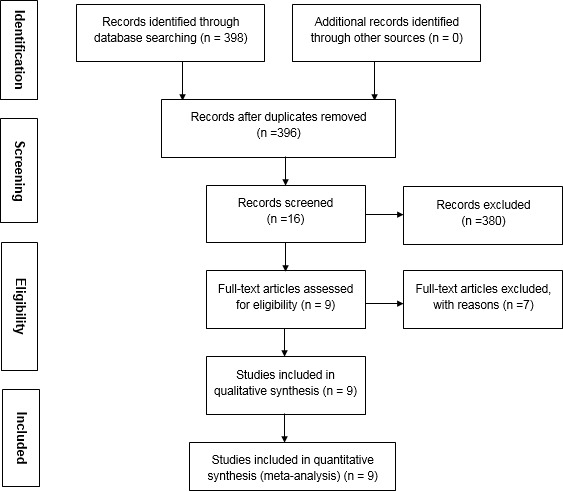
Flow diagram of the study selection process

**Figure 2:**
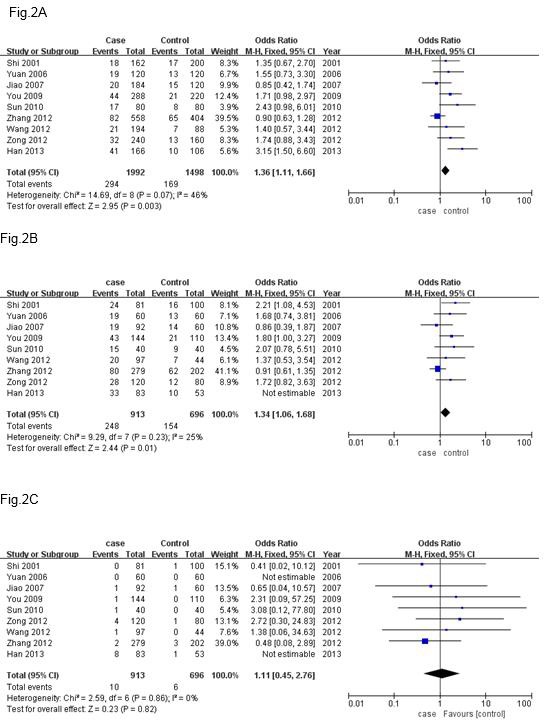
Forest plots for estimation of the association between APOE ε4 allele and depression in different genetic model

A: Allele model; B: Dominant model; C: Recessive model

**Figure 3:**
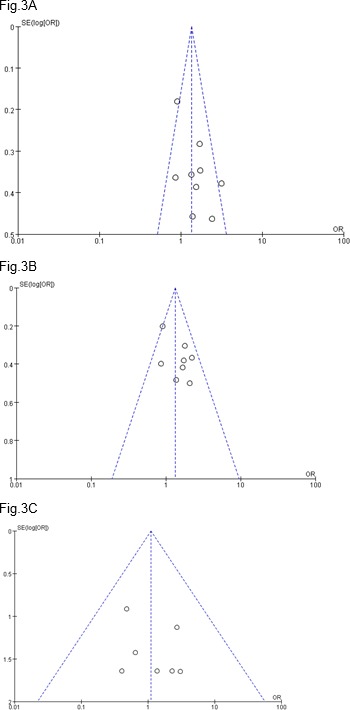
Funnel plots for estimation of the association between APOE ε4 allele and depression in different genetic model

A: Allele model; B: Dominant model; C: Recessive model

**Figure 4:**
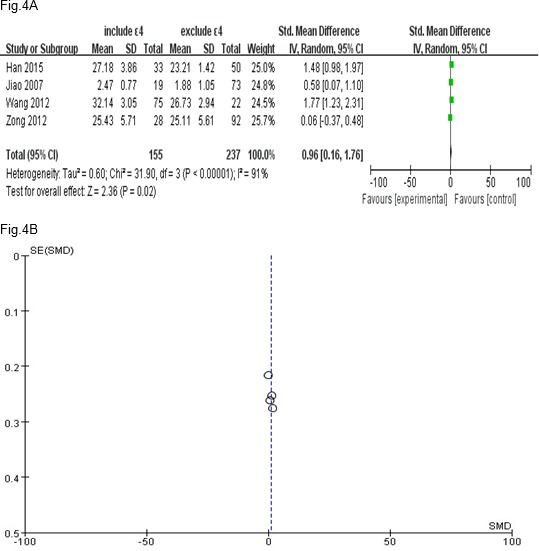
Estimation of the association between APOE ε4 allele and HAMD score in depression

A: Forest plots for the effects of APOE ε4 allele on HAMD score in depression

B: Funnel plots for publication bias, there was no publication bias

**Table 1:** Characteristics of the studies included in this meta-analysis

Author	Year	Ethnicity	Cases						Controls					
			ε2/ε2	ε3/ε3	ε4/ε4	ε2/ε3	ε2/ε4	ε3/ε4	ε2/ε2	ε3/ε3	ε4/ε4	ε2/ε3	ε2/ε4	ε3/ε4
Shi et al (18)	2001	Han	2	55	0	6	1	8	11	69	1	4	0	15
Yuan et al (19)	2006	Han	1	35	0	5	4	15	2	41	0	4	2	11
Jiao et al (20)	2007	Han	1	62	1	10	2	16	0	39	1	7	2	11
You et al (16)	2009	Han	3	76	1	22	11	31	2	55	0	32	6	15
Sun et al (17)	2010	Han	0	21	1	4	3	11	1	26	0	4	1	8
Zhang et al (22)	2012	Han	4	147	2	48	24	54	2	108	3	30	15	44
Zong et al (15)	2012	Han	2	74	4	16	4	20	2	54	1	12	2	9
Wang et al (21)	2012	Han	2	61	1	14	5	14	1	26	0	10	0	7
Han et al (23)	2015	Han	0	41	8	9	1	24	1	36	1	7	1	7

Cases: Patients diagnosed with depression; Controls: Healthy people.

**Table 2:** Information of the Research object included in this meta-analysis

			Cases					Controls			
Author	Year	Diagnostic criteria	Male	Female	Age	Comorbidity	MMSE OR HAMD(SCORE)	Male	Female	Age	HAMD (SCORD)
Shi et al (18)	2001	CCMD-2R	30	51	21-78	No	-	38	62	23-83	-
Yuan et al (19)	2006	DSM-IV&CCMD-3	15	45	60-82	Yes	-	18	42	60-78	<7
Jiao et al (20)	2007	CCMD-3	52	40	18-65	No	HAMD>17	29	31	27-50	-
You et al (16)	2009	DSM-IV	66	78	62-85	No	MMSE>24;HAMD>30	50	60	63-78	<7
Sun et al (17)	2010	DSM-IV&CCMD-3	18	22	60-86	No	MMSE:10-24;HAMD>20	16	24	60-81	-
Zhang et al (22)	2012	DSM-IV&CCMD-3	-	-	15-80	-	HAMD>17	51	151	16-75	<7
Zong et al (15)	2012	DSM-IV	36	84	60-75	no	HAMD>20	25	55	60-74	<8
Wang et al (21)	2012	DSM-IV	33	64	55-78	no	MMSE>24;HAMD>17	21	23	55-78	<7
Han et al (23)	2015	DSM-IV	32	51	43-80	no	HAMD>20	26	27	42-78	<8

Cases: Patients diagnosed with depression; Controls: Healthy people.-: this information was not provided in the primary papers.MMSE OR HAMD (SCORE): Some primary papers provided HAMD (SCORE) only when others provided MMSE (SCORE).Comorbidity: Refers to cardiovascular, cerebrovascular disease and other mental disorders.CCMD: Chinese classification of mental disorders; DSM: Diagnostic and statistical manual of mental disorders.

**Table 3:** Pooled odds ratio for *APOE *ε4 allele in meta-analyses

Gene model	Gene formula	Pooled OR(95%CI)	Z score	*P*-value a	*P*-value b	*P*-value c	*I* ^2^
Allele	**ε4 vs. ε2+ε3**	1.36(1.11-1.66)	2.95	0.003	0.047	0.07	46%
Dominant	**ε4/ε4+ε4/ε3+ε4/ε2 vs. ε2/ε3+ε2/ε2+ε3/ε3**	1.34(1.06-1.68)	2.44	0.001	0.146	0.23	25%
Recessive	**ε4/ε4 vs. ε2/ε2+ε2/ε3ε+ε3/ε3+ε2/ε4+ε3/ε4**	1.11(0.45-2.76)	0.23	0.82	0.874	0.86	0%

OR: odd ratio; CI: confidence interval

a : Fix effect model was used in the meta-analysis to calculate Z score, *P*<0.05 was considered statistically significant.

b : Egger’s test to assess publication bias,* P*<0.05 was considered potential publication bias.

c : Heterogeneity test was assess using Cochran’s (Q)* x*^*2*^ test, *P*<0.01 was considered potential heterogeneity.

The relationship between genetic makeup and susceptibility to depression had long been researched on the basis of family and twin studies. A meta-analysis of seven twin studies on depression shown the heritability impact of depression accounted for 37% while environmental accounting for 63% (5).The tremendous advances in the areas of molecular genetics, biotechnology and sophisticated statistical methods for analyzing complex mode of inheritance created new opportunities to detect so-called susceptibility alleles that increase the risk for depression. *Apolipoprotein E* (*APOE*) included ε2, ε3, and ε4 three alleles might be a susceptibility gene of depression. The molecular basis of the *APOE* gene polymorphisms are differences at two sites in the amino acid sequence (residues 112 and 158). Some have reported that *APOE* gene polymorphisms were a risk factor for triggering depression (6-9). Interestingly, *APOE *ε4 allele impact has been found to differ across ethnic groups. The relative frequencies of the ε2, ε3, and ε4 alleles were 5.3%, 88.4%, and 6.3%, respectively in human (10), the relative frequency of ε4 was the lowest among all ethnic groups (11).

*APOE* gene polymorphism status is hypothesized to be a risk factor for depression (7). However, studies have reached inconsistent conclusions regarding *APOE* polymorphism frequencies in subjects with depression. We performed a meta-analysis of recent studies to assess the relationship between *APOE *ε4 allele and depression.

## Materials and Methods

***Literature search***

The databases PubMed, Web of Science, Wiley Online Library, EMBASE, CBM (Chinese Biomedical Database, http://www.sinomed.ac.cn/), CNKI (Chinese National Knowledge Infrastructure, http://oversea.cnki.net/), VIP (http://www.cqvip.com/), and Wanfang (http://www.wanfangdata.com.cn/) were used to search the literature with the following keywords: (˝Apoprotein E˝ OR ˝apolipoprotein E˝ OR ˝*APOE*˝) AND (˝polymorphism˝ OR ˝mutation˝ OR ˝variant˝) AND depression. We did not impose a language restriction, and we searched the literature published up to February 2018. The most recent results were included when there were multiple publications from the same research group.

***Inclusion and exclusion criteria***

Eligible studies fulfilled the following inclusion criteria: (a) a case-control study design; (b) a focus on the association between *APOE* polymorphisms and depression; (c) the original data, genotype, and allele data are provided; (d) ages of onset < 60, the objects are diagnosed no cognitive impairment (such as Alzheimer’s disease *et al.*) through long-term follow-up studies; and (e) a control group with no history of mental disorders and genotypes in Hardy–Weinberg equilibrium (HWE).

The exclusion criteria were: (a) insufficient information for data extraction, (b) missing information of *APOE* genotype/allele frequency, (c) reviews or case reports.

The data included in this meta-analysis were obtained from the published literature, so written consent by the subjects and ethics committee approval were not required.

***Data extraction***

 According to the inclusion and exclusion criteria, two authors (WW Wang and TH Bao) independently extracted data, including the first author, year of publication, ethnicity, frequencies of *APOE* polymorphisms, and information of research objects in cases and controls. Disagreements on study eligibility were resolved by consulting another author (Ye Ruan).

***Statistical analysis***

The statistical analysis was conducted using Review Manager 5.2 software. A chi-square test was used to test whether the control group conformed to HWE. Differences were considered statistically significant at *P*<0.05.

Heterogeneity between the studies was detected using Cochran’s Q test and *I*^2^ statistics (12), with *P*>0.10 or *I*^2^<50% indicating homogeneity. A fixed effects model was used to calculate the odds ratio (OR) and 95% confidence interval (95% CI). These values were used to determine the association strength between *APOE* polymorphisms and depression. A random effects model was used if *I*^2^>50% (13). In this meta-analysis, we studied on an association between *APOE *ε4 allele and depression, so we used genetic models as that the allele model (ε4 vs. ε2+ε3), the dominant model (ε4/ε4+ε4/ε3+ε4/ε2 vs. ε2/ε3+ε2/ε2+ε3/ε3), and the recessive model (ε4/ε4 vs. ε2/ε2+ε2/ε3ε+ε3/ε3+ε2/ε4+ε3/ε4).

We deleted one study from our meta-analysis at a time to evaluate the potential influences of any single study included in our research. Funnel plots and Egger’s tests were used to assess for possible publication bias. Egger’s test (14) was performed by STATA12.0 software. Potential publication bias was noted when a funnel plot was asymmetric or Egger’s test yielded *P*<0.05.

## Results

***Included studies***

A total of 398 papers were retrieved in a preliminary literature search. After applying our inclusion and exclusion criteria, 9 case–control studies including 903 cases with depression and 737 controls were eligible (15-23). The study selection flow chart is depicted in Figure 1. None of the control groups in this meta-analysis departed from HWE (*P*>0.05). The characteristics of the included papers are presented in Table 1-2.

***APOE ε4 allele was associated with depression***

 In this meta-analysis, an association was detected between *APOE *ε4 allele and depression in three genetic models (allele model, dominant model, and recessive model). The allele and dominant models showed statistically significant differences. There were no statistically significant differences in the recessive model (Table 3, allele: OR=1.36, 95%CI=1.11-1.66, *P*=0.003; dominant: OR=1.34, 95%CI=1.06-1.68, *P*=0.001; recessive: OR= 1.11, 95%CI =0.45-2.76, *P*=0.82). These results showed significant associations between *APOE *ε4 genotype and depression. The Forest plots were presented in 2. 

The fixed effects model was used for the allele, dominant, and recessive models because the heterogeneities were not significant (*I*^2^=46%, 25%, and 0%, respectively). The sensitivity analysis indicated what that deleting one study at a time did not affect the outcomes of the three genetic models.

We did not find any evidence of publication bias in the dominant and recessive models on funnel plots (Figure 3) or by Egger’s tests (*P*=0.047, 0.146, and 0.874, respectively).

***Depression patients carrying APOE*** ***ε*** ***4 genotype got a higher HAMD score***

Depression patients were divided into with-*APOE *ε4 genotype group and without-*APOE*ε4 genotype group. The differences of HAMD score between the two groups were observed. For *I*^2^=91%, the random effects model was used. Only four studies provided HAMD scores of depression patients with and without *APOE* ε4 genotype. It shown depression patients carrying *APOE* ε4 genotype got higher HAMD score, the difference was statistically significant (OR=0.96, 95%CI=0.16-1.76,* P*=0.02). This indicated that *APOE* ε4 genotype associated with severity of depression. The Forest plot was presented in Figure 4A. The publication bias was not seen in Figure 4B.

## Discussion

Depression was a kind of mental disorders with a high recurrence rate that poses serious hazards to human health. The pathogenesis of depression was complex and could be affected by factors such as personality, genetics, and social environments Hypotheses of depression pathogenesis included inflammation, monoamine neurotransmitters and their receptors, hypothalamic-pituitary-adrenal (HPA) axis dysfunction, and the neurotropic factor (24), however the exact pathogenesis’ mechanisms of depression remain unclear.

*APOE* was playing a role in nervous system growth and injury restoration. *APOE* ε2, ε3, and ε4 alleles can combine to yield six genotypes (homozygotes for ε2, ε3, and ε4; heterozygotes for ε2/3, ε2/4, and ε3/4). The frequencies of alleles and genotypes were difference among different ethnic populations and geographic areas (25). Plasma concentrations of total cholesterol and low-density lipoprotein in convalescing depressed patients could predict the risk of suicide during the next depressive episode (26). Low concentrations of cholesterol in serum were associated with depression risk (27). Many depressed patients exhibit neuronal loss and brain structural abnormalities, which might be a result of dyslipidemia (28). Depressed patients had a higher *APOE* ε4 allele frequency, the *APOE* ε4 polymorphism was a risk factor of depression (29-30). Compared with patients who did not carry a ε4 allele, patients with the ε4 allele exhibited more obvious depressive symptoms (31). 

This meta-analysis reviewed the existing eligible studies and examined the association between *APOE *ε4 allele and depression. In this study, 9 papers were included to observant the affection of *APOE* ε4 status on depression. The results shown *APOE *ε4 allele was associated with depression significantly. Depression patients carrying ε4 allele had higher HAMD score. The higher ε4 frequency the more severe of depressive symptoms. This paper reported the association between depression and *APOE *ε4 allele, indicated that *APOE *ε4 allele increased the depression risk, and depressive patients carrying *APOE *ε4 allele had more severe depressive symptoms.

The objects of this study were Han ethnicity, the frequency of the *APOE ε4* allele was 7.5% in Han, which were comparable to values found in other studies of Asian populations (32). The lowest concentration of serum* APOE* was found in the ε4 group (33); ε4 carriers showed increased levels of TNF-α, IL-6, and IL-1β when compared with the ε2 and ε3 carriers in Han population (34). Inflammation and dyslipidemia might be one of hypotheses of depression pathogenesis.

In addition, according to the inclusion and exclusion criteria, only nine eligible articles originated from the Han ethnicity were included in this meta-analysis, it might be the limitations of current study. The truth is always being perfected. When other ethnological data are updated in the future, we will further improve our research on the association of *APOE ε4* allele and depression.

## Conclusion

Collectively, the results of this meta-analysis suggested that *APOE *ε4 allele might be associated with depression, it determined the severity of depression. 

## Conflicts of Interest

The authors declare no conflicts of interest.

## Conflicts of Interest

The funding was obtained from Education Department of Yunnan Province, China (2016ZZX093); Yunnan Provincial Applied Fundamental Research (grant number 2017FE467 (-152); Yunnan Provincial Applied Fundamental Research (grant number 2018FE001 (-148).
